# Non-ST Segment Elevation Myocardial Infarction Secondary to Coronary Multi-Vessel Thrombosis in the Setting of COVID-19

**DOI:** 10.7759/cureus.19258

**Published:** 2021-11-04

**Authors:** Aishwarya Sharma, Abhishek Matta, Danielle Matta, Dinesh Bande

**Affiliations:** 1 Infectious Disease, Sanford Health, Fargo, USA; 2 Hospital Medicine, Sanford Health, Fargo, USA; 3 Internal Medicine, University of North Dakota School of Medicine and Health Sciences, Fargo, USA; 4 Internal Medicine, Sanford Health, Fargo, USA

**Keywords:** sars-cov-2 infection, acute coronary syndrome, multiple thrombi, non-st segment elevation myocardial infarction (nstemi), covid 19

## Abstract

Coronavirus disease 2019 (COVID-19) has been shown to impact the cardiovascular system by causing congestive heart failure, arrhythmias, myocarditis, acute coronary syndrome (ACS), or nonischemic cardiomyopathy. The infection of severe acute respiratory syndrome coronavirus 2 (SARS-CoV-2) triggers the overproduction of proinflammatory cytokines, generating systemic inflammation and a procoagulant state that can lead to cardiovascular morbidity and mortality. Symptomatology may not be discrete with presentation of chest pain, dyspnea, and fatigue, so careful consideration should be applied to cardiovascular complications. Serial troponin dosage as well as EKG changes serve as viable prognosis markers. Prompt dissolution of the thrombi will minimize the extent of the myocardial injury.

## Introduction

On March 12, 2020, the World Health Organization declared coronavirus disease 2019 (COVID-19) as a pandemic. Patients infected with severe acute respiratory syndrome coronavirus 2 (SARS-CoV-2) typically present within two to 14 days following exposure with symptoms of fever, cough, shortness of breath (SOB) and/or pneumonia [1]. Less frequently, COVID-19 has been shown to impact the cardiovascular system by causing congestive heart failure, arrhythmias, myocarditis, acute coronary syndrome (ACS), or nonischemic cardiomyopathy [2]. There are many risk factors that worsen the prognosis of coronavirus disease, one of which is hypertension [2]. We present the case of a 54-year-old male with non-ST segment elevation myocardial infarction (NSTEMI) from 100% occlusive thrombus in the first obtuse marginal artery in the setting of COVID-19 infection.

## Case presentation

A 54-year-old male with a history of hypertension, hyperlipidemia, irritable bowel syndrome, BMI of 32kg/m^2^ and a recent COVID-19 infection presented to our emergency room with chest pain. He was diagnosed with COVID-19 two weeks prior to admission during which he experienced fever, chills, and shortness of breath. He presented to the emergency room with nausea and midsternal chest pain which was sharp and radiated to his back and down his arms bilaterally with an overall pain rating of 8 out of 10. He was afebrile and hemodynamically stable with normal oxygen saturation. He was given topical nitroglycerin and 325mg aspirin. 

**Figure 1:**
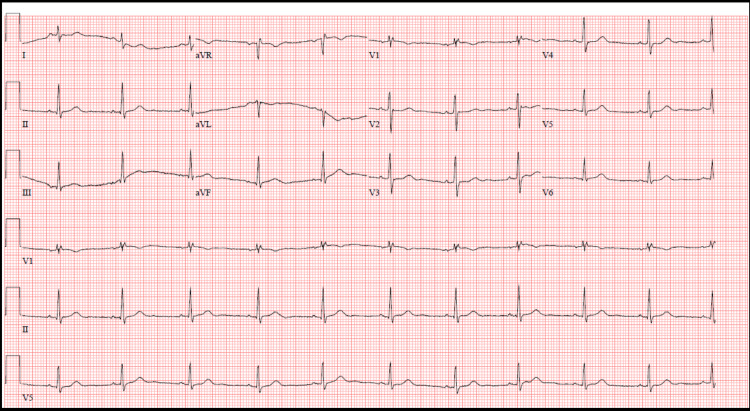
EKG showing normal sinus rhythm and nonspecific ST-T changes

Laboratory findings showed an up-trending troponin I. Rest of the laboratory data is summarized in Table 1. Chest x-ray showed patchy bilateral perihilar infiltrates and linear markings in the retrocardiac space reflecting COVID-19 infection with superimposed atelectasis. EKG showed normal sinus rhythm with nonspecific ST-T changes (Figure 1). Troponin peaked at 26.423ng/ml the following morning.

**Table 1:** Troponin levels trended upwards with a high of 26.423 on day 2. “0 hours” corresponds with the time since admission, etc. Missing values indicate labs were not drawn then.

Laboratory Value	0 hours	3 hours	6 hours	16 hours	Reference Range
Troponin level	0.008	0.142	1.229	26.423	0.000-0.028 ng/mL
B-type Natriuretic Peptide	92				1-100 pg/mL
Hemoglobin	15.5			13.5	13.5-17.5 g/dL
Platelet	494			408	140-400 K/uL
Creatinine	1.17			1.09	0.80-1.30 mg/dL
Creatine Kinase	38				30-200 U/L
C-Reactive Protein	24.1				0.0-8.0 mg/L
Lactate Dehydrogenase	233				125-245 U/L
D-Dimer	0.27				<= 0.49 ug/mL

**Figure 2:**
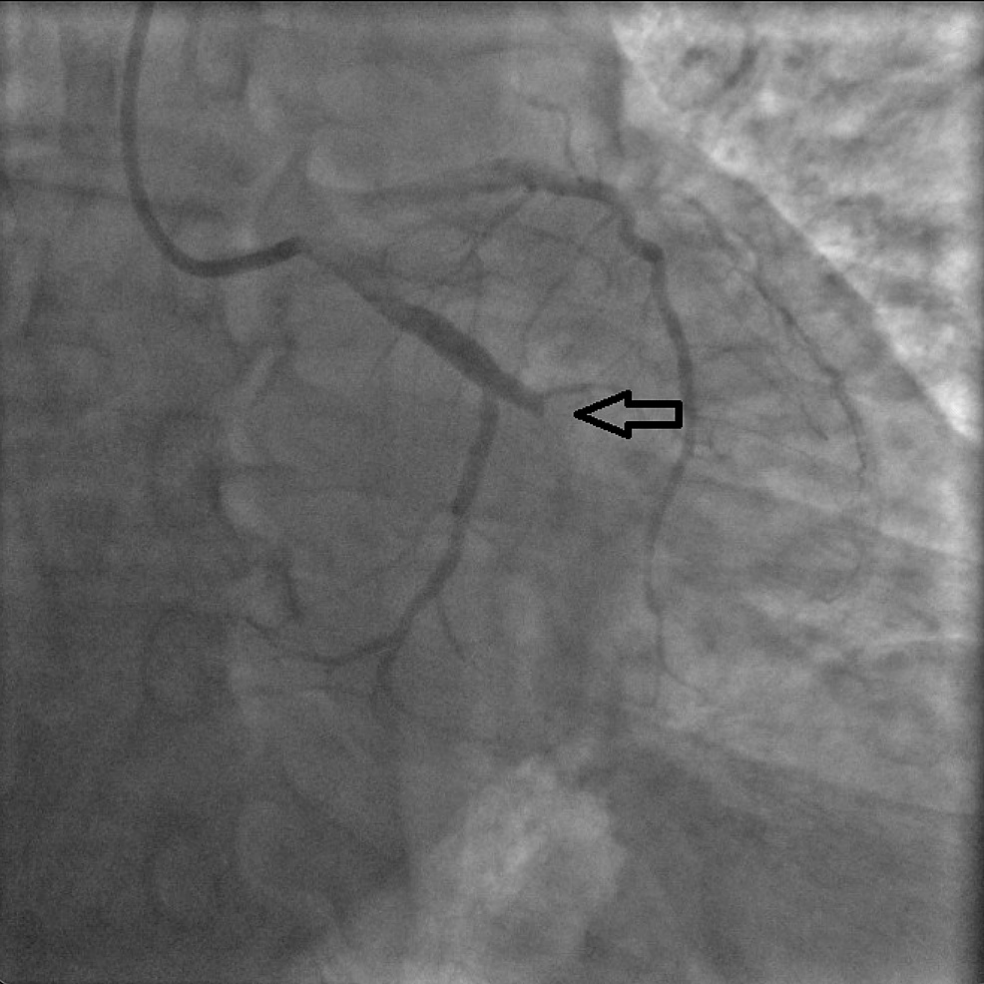
100% obstruction of the first obtuse marginal artery (OM1) by a thrombus

**Figure 3:**
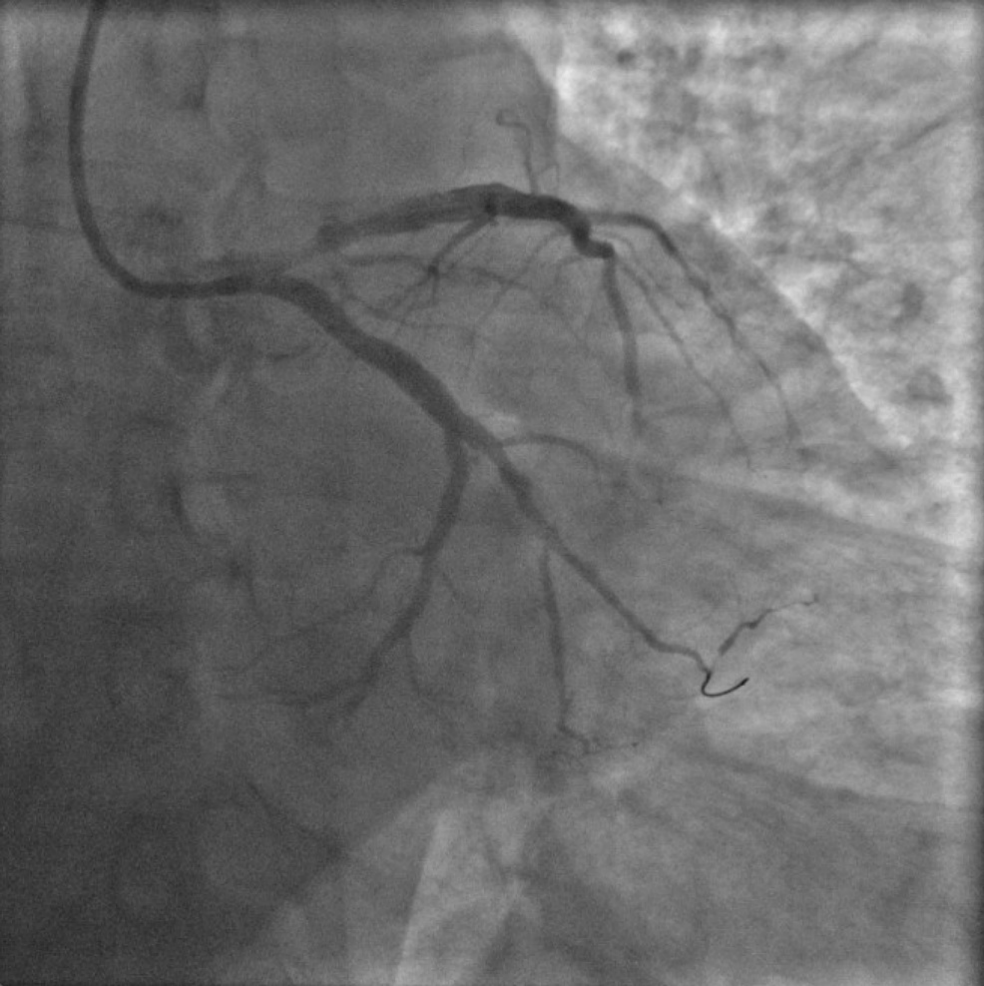
First obtuse marginal artery (OM1) post thrombectomy showing thrombolysis in myocardial infarction (TIMI) 3 flow

**Figure 4:**
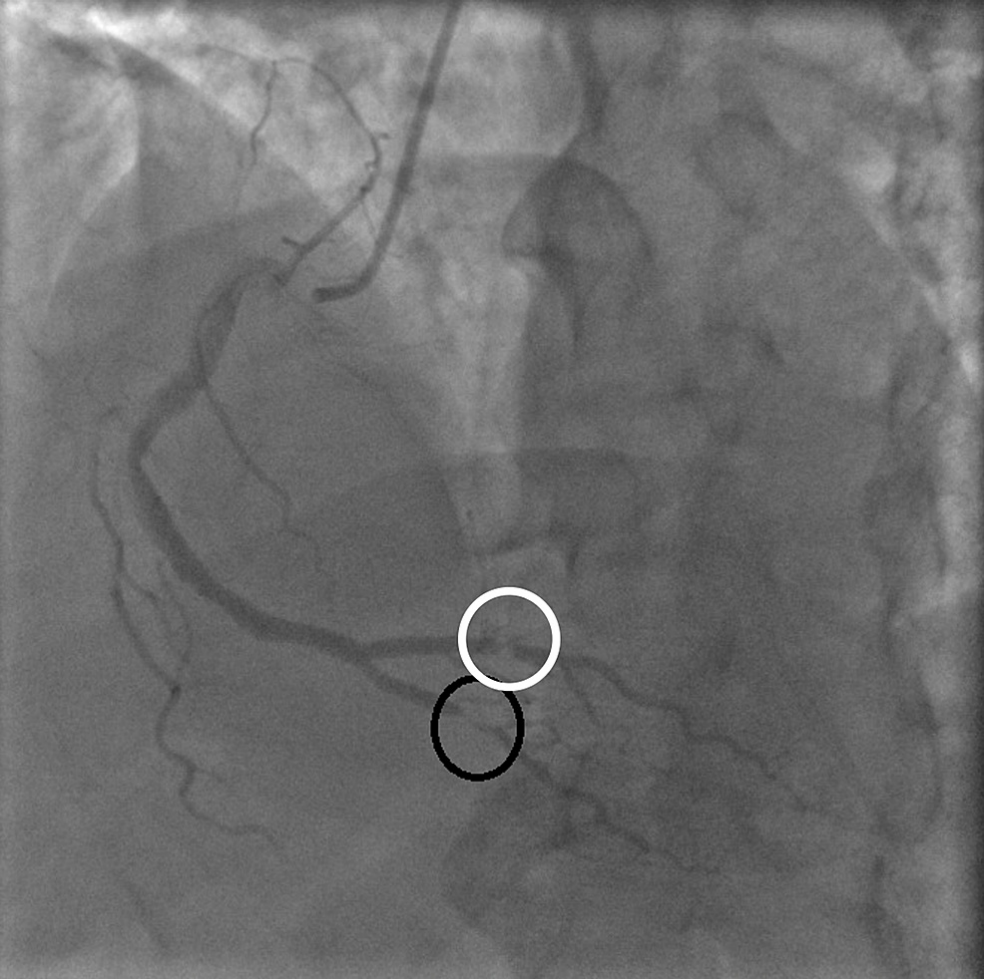
Thrombus causing 80% obstruction of right posterior descending artery (black circle) and another thrombus causing 80% obstruction in right posterolateral artery (white circle)

The patient's medication history included lisinopril 10mg daily which was continued during the hospital stay, trazodone 50mg at bedtime for insomnia, citalopram 40mg daily, lorazepam 0.5mg daily as needed for anxiety. He was started on IV heparin, 81mg aspirin daily, 12.5mg metoprolol twice a day, and 80mg atorvastatin daily. Echocardiogram showed a normal ejection fraction of 60% with no regional wall abnormalities. The patient was loaded with 300mg clopidogrel before the coronary angiogram on day 2 which was significant for 100% occlusion of the first obtuse marginal artery (OM1) by a thrombus (Figures 2, 3). In addition, the right posterolateral artery and the right posterior descending artery were both 80% occluded by thrombi (Figure 4). Since these occlusions were from thrombi without the presence of the underlying plaque, the etiology of the thrombi is likely secondary to a procoagulant state in the setting of COVID-19 rather than from atherosclerosis. A successful thrombectomy and balloon angioplasty were completed in the first obtuse marginal artery. Post-angiogram, the patient experienced some premature ventricular complex (PVC) on telemetry which resolved following an increased dosage of metoprolol to 25mg twice a day (bid). He was discharged on day 4 on aspirin 81mg daily for one month, continuing clopidogrel 75mg daily, metoprolol 25mg bid, and started on apixaban 10mg bid for seven days followed by 5mg bid. He followed up with cardiology in the clinic after four months. Stress echo was performed to assess for any significant atherosclerosis and didn’t show any concern for inducible ischemia. Apixaban was stopped at this point. No other issues were noted on further follow-up.

## Discussion

COVID-19 can present clinically with a wide range of symptoms from asymptomatic to characteristic upper respiratory tract symptoms in the first week to dyspnea, cough, and hypoxemia after day 10 [3,4]. Although COVID-19 classically presents with respiratory symptoms, thrombosis following a COVID-19 infection can present as a pulmonary embolism, cerebral infarction, and less commonly as acute myocardial infarction [5,6]. It is believed that SARS-CoV-2 infects angiotensin converting enzyme-2 (ACE-2) receptors on endothelial cells which results in the accumulation of inflammatory cells and subsequent complement-mediated microvascular injury [7]. The systemic inflammatory response triggers a cytokine storm which results in endothelial damage and upregulation of tissue factor followed by activation of the coagulation cascade [8]. As a result, a hypercoagulable state is generated which can precipitate thromboembolic events. 

Presence of cardiovascular risk factors such as hypertension and obesity can worsen cardiovascular morbidity. Hypertension can also lead to a pro-inflammatory state which can worsen the prothrombotic state [2]. ACS presentation can be masked by respiratory symptoms of viral pneumonia, so timely diagnosis is reliant on careful monitoring. Clinical manifestations may include anginal chest pain, dyspnea, syncope, dizziness, nausea, and fatigue [9]. Elevated troponin, EKG presentation of ST elevation and/or Q waves, and imaging studies aid in the prognosis and diagnosis of ACS in confirmed COVID-19 patients [9]. Delayed presentation and diagnosis of myocardial infarction (MI) can result in free wall rupture, ventricular septal defect, and left ventricular aneurysms. Standard treatment for an ACS such as percutaneous coronary intervention (PCI)-mediated reperfusion therapy followed by aspirin, P2Y12 inhibitor, beta blocker and statin should be administered [10]. Anticoagulation can be considered in patients with future risk of thrombi formation. 

Our patient has been experiencing COVID-19 symptoms for almost two weeks and presented with characteristic symptoms of angina. Troponin gradually trended up, suggestive of NSTEMI. Coronary angiogram showed thrombosis in multiple vessels but no significant atherosclerotic burden. This points towards the NSTEMI being secondary to a procoagulant state and intra-arterial thrombi secondary to COVID-19 rather than atherosclerotic plaque rupture. Only thrombectomy was performed in our patient due to the absence of atherosclerotic disease which usually requires drug-eluting stent placement. We started the patient on anticoagulation medication based on the prevailing guidelines at the time. Repeat stress test four months after the thrombectomy didn’t show any inducible ischemia. This further supports the absence of atherosclerotic burden in our patient. 

## Conclusions

ACS is a potential complication of COVID-19 due to the subsequent procoagulant state and systemic inflammation. Patients present with characteristic symptoms of cardiac ischemia including anginal chest pain, dyspnea and nausea. Up trending troponin levels and standard EKG changes serve are important in making the diagnosis. Thrombi may be noted in the coronary arteries on imaging rather than atherosclerosis. Prompt reperfusion therapy via PCI followed by a pharmacological regimen of aspirin, P2Y12 inhibitor, beta blocker and statin is necessary to limit the extent of myocardial injury. A short course of anticoagulant therapy should be considered in patients at high risk of thrombosis.
